# Reconsidering the male disadvantage in bronchopulmonary dysplasia: three exceptions

**DOI:** 10.1038/s41390-025-04238-z

**Published:** 2025-06-16

**Authors:** Theodore Dassios, Charles C. Roehr

**Affiliations:** 1 Department of Women and Children’s Health, Faculty of Life Sciences and Medicine, King’s College London, London, UK; 2 Neonatal Intensive Care Unit, University of Patras, Patras, Greece; 3 National Perinatal Epidemiology Unit, Oxford Population Health, University of Oxford, Oxford, UK; 4 Newborn Care, Southmead Hospital, Women’s and Children’s Division, North Bristol NHS Trust, Bristol, UK; 5 Faculty of Health and Life Sciences, University of Bristol, Bristol, UK

The established paradigm in the epidemiology of bronchopulmonary dysplasia (BPD) includes a clear male dominance in chronic respiratory morbidity which has been firmly established through large multicenter epidemiological studies and meta-analyses.^1^ That male preterm infants fare worse compared to their female counterparts of similar gestation is an observation which extends beyond BPD and encompasses other important neonatal outcomes of prematurity such as mortality and major cranial hemorrhage or ischemic lesion.^2^ The male disadvantage following premature birth persists throughout childhood, as male infants suffer from more neurodevelopmental impairment including cognitive delay, more frequent use of inhalers and, overall, are higher health-care utilizers compared to their female counterparts.^2^ The exact pathophysiological background behind this phenomenon is not completely understood but is thought to be related to a variety of intrauterine mechanisms such as a slower rate of maturation in male compared to female fetuses, the uterus being a less hospitable immunological environment for male fetuses and the differential effect of prenatal sex steroids on the intrauterine environment and the developing fetus.^3^

In this issue, van Westering-Kroon and co-workers have undertaken a meta-analysis on the risk of developing BPD in male compared to female newborn infants.^4^ The authors have undertaken an exhaustive literature search, included 541,826 infants from 222 studies and applied a novel Bayesian model-averaged statistical approach in their meta-analysis. This statistical method can help differentiate between the absence of evidence and the evidence of no effect, unlike the more commonly used null hypothesis significance testing. Using this approach, they compared male and female infants by testing different possible definitions of BPD, including the diagnosis at 28 days or 36 weeks postmenstrual age, the categories of mild, moderate and severe and the subgroups of infants below 27, 25, and 23 weeks of gestational age. They further investigated sex differences in BPD in relation to potential temporal changes, geographical variation and the diagnosis of BPD-associated pulmonary hypertension.^4^

As expected, the authors have confirmed the previously well-described, cardinal finding of male dominance in BPD using this novel methodology and reported that the risk of developing BPD is approximately 20% higher in male than in female infants. Furthermore, the authors have also approached three pertinent and not previously well-described sub-questions, and have thus formulated three distinct and clinically interesting exceptions to the general rule of male dominance in BPD:

1. **Not in mild BPD:** The male predominance seems to be absent in the category of mild BPD which is defined as the need for oxygen or respiratory support at 28 days of postnatal life, but is no longer present at 36 weeks of postmenstrual age, at which point these infants are breathing unassisted in room air. That a diagnosis of mild BPD is of a weaker significance, reflects the changing pathophysiology and epidemiology of BPD, as the most immature infants will naturally need some respiratory support in the first postnatal weeks, without necessarily requiring such support by the time point of 36 weeks postmenstrual age, which for them comes considerably later. There has been indeed some skepticism around the clinical utility of the category of mild BPD in the current era, as this diagnosis has overall limited discriminatory capacity and can poorly identify infants at risk for subsequent clinical complications. At the epidemiological level it has been suggested that mild BPD can potentially be grouped together with “no BPD”.^5^
2. **Not below 25 weeks of gestational age:** The loss of male dominance in severe BPD below the gestational age threshold of 25 weeks is also an important finding, which agrees with recent whole population data reflecting the current care of these very frail and immature infants.^6^ The biological basis of this phenomenon has not been fully elucidated but could potentially be related to the lack of expression of both male and female sex hormones at very low gestations, which would be associated with impaired surfactant production in the case of male hormones like dihydrotestosterone, and the loss of the protective effect of progesterone and estradiol which enhance the reabsorption of alveolar fluid at birth.^7^ It is also possible that in the meta-analysis of van Westering-Kroon and co-workers, sex differences were simply not strong enough to be detected in preterm infants of less than 25 weeks, as the risk for BPD or death is already nearly 90% in these infants^8^ and the impact of immaturity possibly overshadows any other potential contributing factor. Irrespective of the mechanism, this knowledge constitutes useful clinical information as it can assist with individualized prognosis and counseling.
3. **Not in pulmonary hypertension:** Although the development of pulmonary hypertension in the context of BPD is clearly important and associated with significantly higher mortality and morbidity,^9^ the male disadvantage was also absent for this complication in the meta-analysis by van Westering-Kroon and co-workers. The authors note that their results suggest that females are less likely to develop moderate to severe BPD but may be equally susceptible as males to develop what has been described as the “vascular phenotype” of BPD. This finding might be explained by the later time window during which pulmonary hypertension develops in the context of BPD and might also be related to an increased risk for impaired pulmonary vascular development in the female fetuses.

The authors made two further epidemiological remarks which, however valuable, might need to be seen in the light of some inherent methodological limitations of conducting a meta-analysis and the reliability of the corresponding data from the included studies. The authors reported that sex differences in BPD have not changed over time, despite the increased survival of more immature infants in whom these differences are less profound. This observation should be read in the context of the changes in the treatment and utilized definitions of BPD over the recent years, and the possibly diverging actions of these parameters on male and female infants. For example, one might speculate that more immature infants survive in the more recent years and in these gestations there is less male disadvantage, while on the same time there is a decrease in sex differences in mortality with boys being more susceptible than girls to oxidative stress-related complications.^10^ Similarly, it was interesting to learn that the evidence for male disadvantage in BPD was particularly strong in the North American and European populations and in the countries with higher sociodemographic characteristics, but not as strong in East Asia and inconclusive in Latin America and Oceania. As the authors discuss, these results might need to be evaluated in the light of data completeness and numerous other factors which could explain such variations, such as regional genetic and environmental differences, and disparities in lifestyle, clinical practices, and the financial capacities of different health systems. All these parameters might influence how transferrable these conclusions are in the setting of the European or North American populations and healthcare systems. These regional discrepancies, however, certainly deserve further study, since possible interventions in populations with a higher incidence of the disease might bring about a positive change of a greater magnitude.

In conclusion, the meta-analysis of van Westering-Kroon and co-workers has applied a novel methodological approach, which reduces the risk of misinterpretation of the traditional null hypothesis testing approach, to confirm the male disadvantage in BPD following preterm birth. This male predominance in chronic preterm respiratory morbidity has three distinct and notable exceptions: not in mild BPD, not below 25 weeks of gestation and not in BPD-associated pulmonary hypertension.
